# Morphological alterations of exogenous surfactant inhibited by meconium can be prevented by dextran

**DOI:** 10.1186/1465-9921-7-86

**Published:** 2006-06-06

**Authors:** Matthias Ochs, Markus Schüttler, Guido Stichtenoth, Egbert Herting

**Affiliations:** 1 Institute of Anatomy, Experimental Morphology, University of Bern, Baltzerstrasse 2, CH-3012 Bern, Switzerland; 2 Department of Anatomy, Division of Electron Microscopy, University of Göttingen, Kreuzbergring 36, D-37075 Göttingen, Germany; 3 Department of Pediatrics, University of Lübeck, Ratzeburger Allee 160, D-23538 Lübeck, Germany

## Abstract

**Background:**

Surfactant dysfunction due to inhibition is involved in the pathophysiology of meconium aspiration syndrome. Dextran addition has been shown to reverse exogenous surfactant inactivation by meconium, but the precise mechanisms and the morphological correlate of this effect are yet unknown. Morphological surfactant analysis by transmission electron microscopy (TEM) and stereology allows the differentiation of active (large aggregates = LA) and inactive (small aggregates = SA) subtypes.

**Methods:**

To determine the in vitro effects of meconium and dextran addition on the morphology of a modified porcine natural surfactant (Curosurf), Curosurf samples were either incubated alone or together with meconium or with meconium and dextran, fixed and processed for TEM. Volume fractions of surfactant subtypes [lamellar body-like forms (LBL), multilamellar vesicles (MV), unilamellar vesicles (UV)] were determined stereologically.

**Results:**

All preparations contained LBL and MV (corresponding to LA) as well as UV (corresponding to SA). The volume fraction of UV increased with addition of meconium and decreased with further addition of dextran. Correspondingly, the UV/(LBL+MV) ratio (resembling the SA/LA ratio) increased when meconium was added and decreased when dextran was added to the surfactant-meconium mixture.

**Conclusion:**

Meconium causes alterations in the ultrastructural composition of Curosurf that can be visualized and analyzed by TEM and stereology. These alterations resemble an increase in the SA/LA ratio and are paralleled by an increase in minimum surface tension. Dextran prevents these effects and may therefore be a useful additive to exogenous surfactant preparations to preserve their structural and functional integrity, thereby improving their resistance to inactivation.

## Background

The surfactant system of the lung prevents alveolar collapse by reducing alveolar surface tension. Growing evidence suggests that certain surfactant components also have immunomodulatory functions. Surfactant, synthesized and secreted by type II alveolar epithelial cells, is a complex mixture of lipids, mainly saturated phospholipids, and proteins, among them the surfactant apoproteins SP-A, -B, -C and -D [1]. The two hydrophilic, large molecular weight surfactant apoproteins SP-A and SP-D are lost during extraction with organic solvents used in the production of the commercially available surfactant preparations of bovine or porcine origin. The glycoproteins SP-A and SP-D are members of the collectin family and seem to "orchestrate the lung's immune response" [2,3]. In contrast, the hydrophobic surfactant proteins SP-B and SP-C are contained in natural modified surfactants for clinical use. SP-B and SP-C are essential for the biophysical activity of surfactant preparations [4,5].

Intraalveolar surfactant is present in different morphological forms which, according to current models, correspond to different stages within surfactant metabolism [6]. Surfactant material is secreted by type II cells as lamellar bodies. Within the hypophase of the alveolar lining layer, lamellar body-like forms undergo transformation into tubular myelin figures when SP-A is added to their outer lamellae [7]. Tubular myelin is thought to be the precursor of material that is inserted into the surface film. "Spent" surfactant is found in the hypophase as unilamellar vesicles and can be taken up by type II cells. After differential centrifugation of intraalveolar surfactant material obtained by bronchoalveolar lavage, surface active large aggregates (LA), largely corresponding to lamellar body-like forms, tubular myelin and multilamellar vesicles, and inactive small aggregates (SA), largely corresponding to unilamellar vesicles, can be distinguished [8-10].

Several studies indicate that dysfunction of pulmonary surfactant due to inhibition plays a key role in the pathophysiology of meconium aspiration syndrome [11-15]. Thus, there is a rationale for exogenous surfactant therapy in neonates with severe respiratory failure due to meconium aspiration [16-21]. In vitro, meconium inhibits exogenous surfactant preparations in a dose-dependent manner [22]and alters exogenous surfactant morphology [23]. The addition of nonionic polymers like dextran has been shown to reverse the inactivation of exogenous surfactant by meconium in vitro [24-26]. This and other in vitro and in vivo findings [27-30]have led to the concept that surfactant inactivation in various forms of acute lung injury may be overcome with the help of nonionic polymers [31]. However, the precise mechanisms and the morphological correlate of these effects are yet unknown.

Inactivation of intraalveolar surfactant in acute lung injury and related conditions includes increased conversion of surface active LA to SA with poor surface activity. The SA/LA ratio of surfactant material obtained by bronchoalveolar lavage is therefore used to assess the biophysical activity of the endogenous surfactant system [8,10,32]. These two different functional states within surfactant metabolism correspond to different surfactant subtypes that can be distinguished morphologically at the electron microscopical level. Using a transmission electron microscopical and stereological approach, active and inactive intraalveolar surfactant subtypes can be analyzed qualitatively and quantitatively in their natural localization and microorganization within the lung. Ultrastructural alterations resembling an increase in the SA/LA ratio have been demonstrated in various forms of acute lung injury [33-35]. A stereological approach has also been used to analyze exogenous surfactant preparations [36,37], but so far, it has not been applied to investigate meconium inhibition of exogenous surfactants.

Therefore, the aim of the present study was to analyze the in vitro effects of meconium and dextran addition on the morphology of a modified porcine natural surfactant (Curosurf) by means of transmission electron microscopy and to quantitate active and inactive subtypes by stereology.

## Methods

### Meconium, surfactant, dextran

Meconium from 16 healthy term neonates was collected, lyophilised and pooled following parental consent and approval of the study by the local ethics committee. 49.4 g of the material had a dry weight of 12.2 g (i.e. water content: 75.3%) following lyophylization. For our studies all materials were resuspended/diluted in saline containing 1.5 mmol/l CaCl_2 _to reach a meconium concentration of 0.04 or 0.6 mg/ml (wt/vol), a surfactant (Curosurf, Batch No.: 194/09 original concentration: 80 mg/ml, a kind gift from Nycomed Pharma, Unterschleissheim, Germany) concentration of 10 mg/ml and a dextran (Dextran T 500 (MW 500,000 Da), Pharmacia LKB, Uppsala, Sweden) concentration of 10 mg/ml or 20 mg/ml, respectively. For the final experiments the different components were mixed, thereby reaching final concentrations of 2.5 mg/ml surfactant, 0.02 or 0.3 mg/ml meconium and 5 or 10 mg/ml of dextran, respectively.

### Fixation and processing for electron microscopy

Curosurf samples (2.5 mg/ml) were either incubated alone or together with meconium or with meconium and dextran for 30 min at 37°C before analysis. Samples containing Curosurf (C), Curosurf + meconium 0.02 mg/ml (CM0.02), Curosurf + meconium 0.3 mg/ml (CM0.3), Curosurf + meconium 0.3 mg/ml + dextran 5 mg/ml (CM0.3D5), and Curosurf + meconium 0.3 mg/ml + dextran 10 mg/ml (CM0.3D10) were fixed and processed for transmission electron microscopy.

Samples of 0.2 ml were fixed in an equal volume of 1.5% glutaraldehyde and 1.5% formaldehyde (made from freshly depolymerized paraformaldehyde) in 0.15 M Hepes buffer, pelleted by centrifugation with a 5417/R centrifuge (Eppendorf, Hamburg, Germany) at 10,600 g for 10 min, postfixed in 1% OsO_4 _in 0.1 M sodium cacodylate, bloc-stained in half-saturated aqueous uranyl acetate over night, dehydrated in an ascending series of acetone and embedded in araldite [see [38]].

Ultrathin sections from 2–3 samples per group were counterstained with lead citrate and analyzed qualitatively and quantitatively at an EM 900 transmission electron microscope (Leo, Oberkochen, Germany) at an accelerating voltage of 50 kV.

### Stereological analysis

Volume fractions of surfactant subtypes [lamellar body-like forms (LBL), multilamellar vesicles (MV), unilamellar vesicles (UV)] were determined stereologically by point counting in systematic uniform random test fields distributed over the whole section. Between 100 and 200 counting events per ultrathin section were generated to ensure that the total observed variability was dominated by the biological variability among samples and not by the variability among stereological measurements within one sample [39].

### Surface tension measurements

Surface tension was determined in vitro using a pulsating bubble [40]surfactometer (Electronetics corporation, Buffalo, New York). In short, a small volume of fluid was transferred into a sample chamber and an air bubble was created. During a 30 sec adsorption period, the surface active material forms a film at the air-liquid interface. Pulsation is started at a frequency of 20 cycles/min by which the bubble undergoes cyclic area compression (50 % of the surface area). Surface tension at minimum bubble size (γ_min_) after 5 min of pulsation at a temperature of 37°C is calculated by a computer program from the preset bubble radius and the recorded pressure across the bubble wall making use of the law of Laplace [40].

## Results

### Electron microscopy

**Figure 1:**
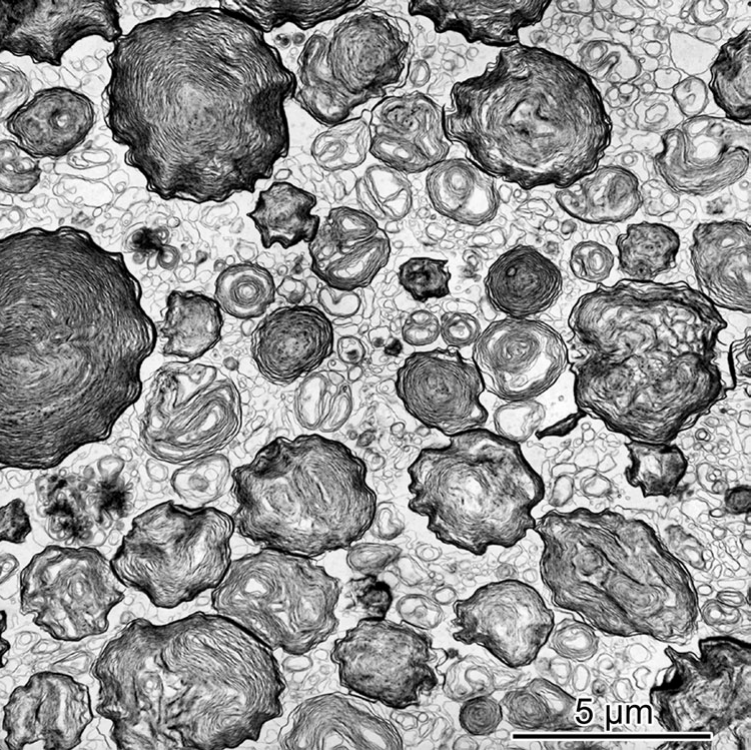
**Ultrastructure of Curosurf**. Transmission electron micrograph from a pure Curosurf preparation. For details, see Materials and Methods. The preparations are composed of lamellar body-like forms (LBL), multilamellar vesicles (MV), and unilamellar vesicles (UV).

Since the biochemical composition of endogenous surfactant and commercially available exogenous surfactant preparations is different [36], there are also morphological differences between intraalveolar surfactant subtypes and exogenous surfactants. Due to the lack of SP-A, exogenous surfactants do not contain tubular myelin. Other surfactant subtypes, however, were found in Curosurf that can be compared to endogenous surfactant subtypes. Three morphologically different subtypes could be distinguished in Curosurf samples: lamellar body-like forms (LBL), multilamellar vesicles (MV), and unilamellar vesicles (UV) (Fig. 1A).

**Figure 2:**
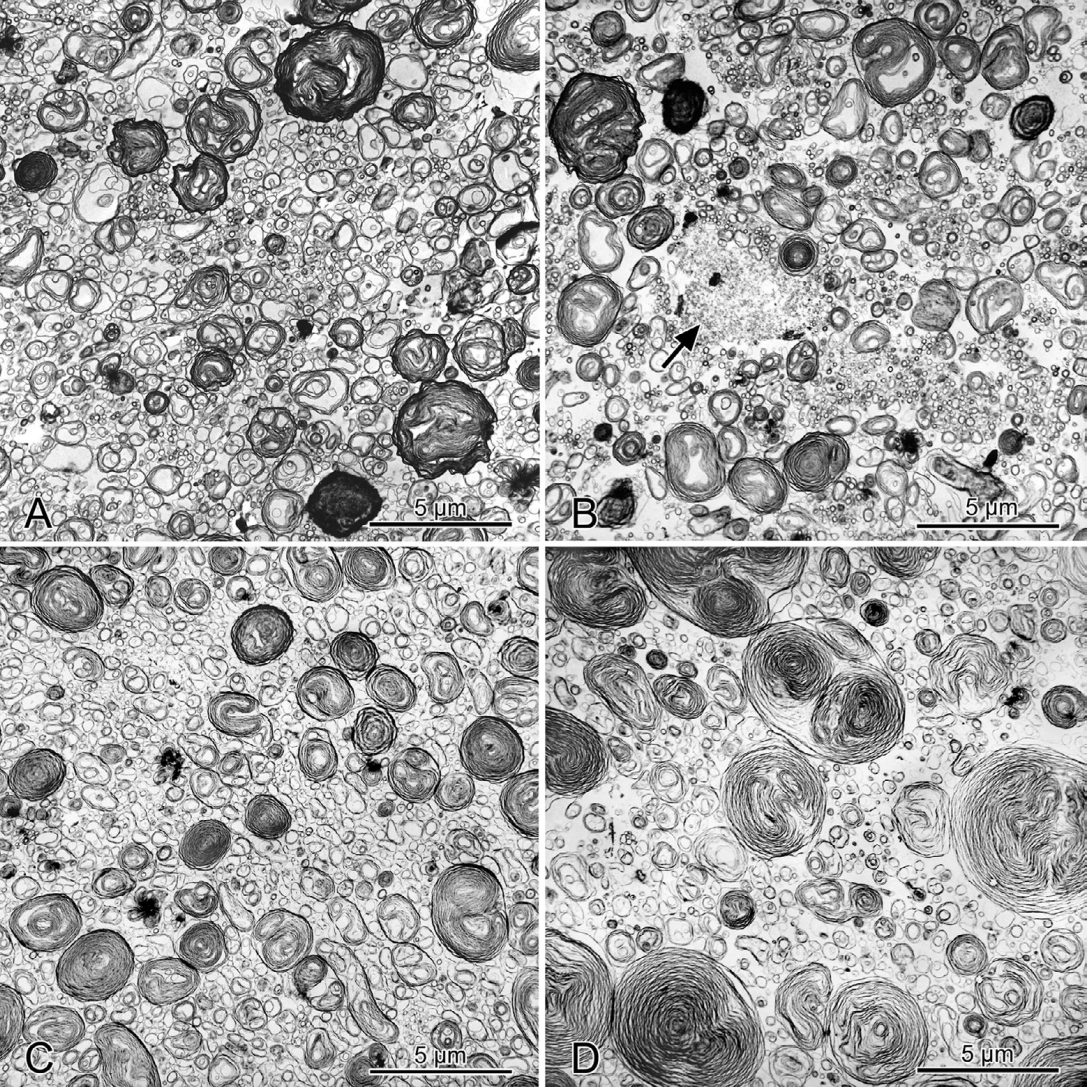
**Ultrastructure of Curosurf, meconium, and dextran**. Transmission electron micrographs from Curosurf preparations after incubation with 0.02 mg/ml meconium (**A**), 0.3 mg/ml meconium (**B**), 0.3 mg/ml meconium + 5 mg/ml dextran (**C**), and 0.3 mg/ml meconium + 10 mg/ml dextran (**D**). With increasing concentrations of meconium, more unilamellar vesicles become visible (**A **and **B**). The flocculent material visible in the center of **B **most likely represents meconium material (arrow). The addition of increasing concentrations of dextran leads to more lamellar body like forms and less unilamellar vesicles (**C **and **D**).

All Curosurf-meconium preparations with or without dextran contained LBL, MV and UV, however in varying amounts (Fig. 2A–D). In accordance with previous studies on intraalveolar surfactant ultrastructure [34]and current models of surfactant metabolism [8-10], we classified LBL and MV as LA and UV as SA for subsequent stereological analysis.

### Stereology

**Figure 3:**
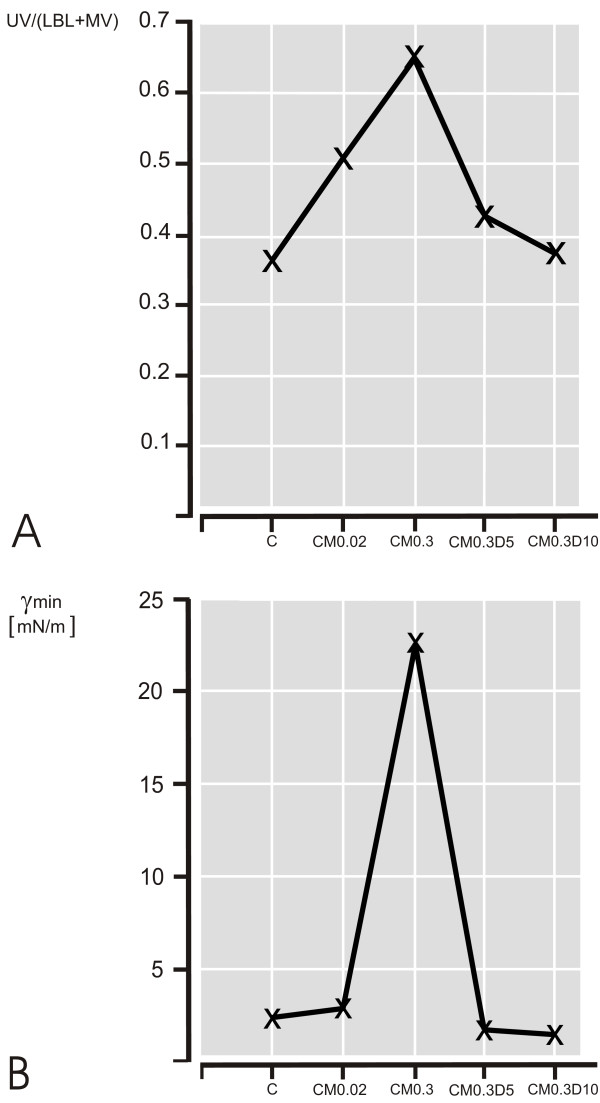
**Stereology versus minimum surface tension**. **A**: Ratio of unilamellar vesicles to lamellar body-like forms and multilamellar vesicles, used as a morphological correlate to the small to large aggregate ratio. Samples contained Curosurf (C), Curosurf + meconium 0.02 mg/ml (CM0.02), Curosurf + meconium 0.3 mg/ml (CM0.3), Curosurf + meconium 0.3 mg/ml + dextran 5 mg/ml (CM0.3D5), and Curosurf + meconium 0.3 mg/ml + dextran 10 mg/ml (CM0.3D10). **B**: Minimum surface tension of Curosurf, Curosurf/meconium- and Curosurf/meconium/dextran-mixtures as measured after 5 min of cyclic area changes in the pulsating bubble surfactometer. Values are means from 5 repeated measurements.

The stereological results are summarized in Table 1 and illustrated in Figure 3A. The volume fraction of UV increased with addition of meconium (C: 27%; CM0.02: 34%; CM0.3: 39%) and decreased to control values with further addition of dextran (CM0.3D5: 30%; CM0.3D10: 28%). Correspondingly, the UV/(LBL+MV) ratio (resembling the SA/LA ratio) increased when meconium was added (C: 0.37; CM0.02: 0.51; CM0.3: 0.65) and decreased to control values when dextran was added to the surfactant-meconium mixture (CM0.3D5: 0.43; CM0.3D10: 0.38).

**Table 1:** Summarized stereological results

Preparation	V_V_(LBL) [%]	V_V_(MV) [%]	V_V_(UV) [%]	UV/(LBL+MV)
C	26.4	46.4	27.2	0.37
CM0.02	33.1	33.1	33.9	0.51
CM0.3	38.0	22.7	39.2	0.65
CM0.3D5	42.4	27.3	30.2	0.43
CM0.3D10	43.1	29.2	27.7	0.38

Abbreviations: V_V _= volume fraction, LBL = lamellar body-like forms, MV = multilamellar vesicles, UV = unilamellar vesicles, C = Curosurf, M = meconium, D = dextran.

### Surface tension measurements

The changes in biophysical activity, as measured by minimum surface tension, are depicted in Figure 3B. Surfactant in the absence of meconium reaches minimum surface tension below 5 mN/m. Addition of meconium inactivates the surface active material and causes an immediate rise in minimum surface tension > 20 mN/m. In parallel to the findings observed in the stereological analysis, the addition of dextran restored the surface activity of Curosurf. This phenomenon could not only be observed following coincubation of Curosurf, meconium and dextran. The addition of dextran to inactivated samples actually reversed the inhibitory effects of meconium in a meconium/surfactant mixture with previously disturbed biophysical function.

## Discussion

The development of exogenous surfactant therapy for the treatment of respiratory distress syndrome (RDS) in premature babies with primary surfactant deficiency is considered one of the major advances in neonatology in our time [1,41,42]. Based on this success, the indications for exogenous surfactant therapy have widened to prevent or treat respiratory failure caused by impairment of an originally intact surfactant system like the acute respiratory distress syndrome (ARDS) and other "non-RDS" respiratory disorders [8,16,19,32,41,43]. In these cases, however, the efficacy of exogenous surfactant therapy depends largely on the ability of the surfactant preparation to resist the inactivation that caused alterations of the endogenous surfactant system [1,17,41].

The present in vitro study demonstrates that meconium causes alterations in the ultrastructural composition of the modified porcine natural surfactant Curosurf that can be visualized and analyzed qualitatively and quantitatively using transmission electron microscopy and stereological methods. Incubation of Curosurf with increasing concentrations of meconium results in an increase in the volume fraction of unilamellar vesicles and a decrease in the volume fraction of multilamellar vesicles (resembling an increase in the SA/LA ratio), which could be due to relative changes in particle number and/or mean particle size. This ultrastructural finding is paralleled by an increase in minimum surface tension. This demonstrates that the morphological alterations observed in the present in vitro study are relevant to biophysical surfactant function.

An increased SA/LA ratio, as demonstrated by morphological criteria in the present study, is known to be present in various forms of acute lung injury [8,15,32]. In general, surfactant can be inactivated by biophysical (e.g. by competition with surfactant lipids at the air-liquid interface) or biochemical (e.g. by enzymatic degradation) mechanisms. In meconium-induced surfactant inactivation, inhibitors of surfactant function like plasma proteins [4]or bilirubin [44]seem to be involved [18,19,43]. Meconium also contains secreted phospholipase A_2_[45], so that enzymatic degradation of lipids and proteins is another inhibitory mechanism to be discussed. Recently, Kakinuma et al. reported an accelerated subtype conversion as an additional mechanism for meconium-induced surfactant inactivation [15], indicating that also the sensitive interaction between surfactant lipids and proteins that is needed for the formation of the surface active film can be disturbed by meconium.

The mechanisms by which dextran counteracts the inhibitory effects of meconium on surfactant function are as yet unclear [24]. It is possible that the addition of hydrophilic polymers lowers the energy barrier to surfactant adsorption via a nonspecific depletion attraction [46]. In our study, dextran prevents an increased conversion of LA to SA, thereby exhibiting a protective/stabilising effect on LA. A similar function has been described in vivo for SP-A [47,48]. Moreover, SP-A inhibits surfactant phospholipid hydrolysis by secreted phospholipase A_2_[49]. The idea to look for functional analogues for the carbohydrate binding domain of SP-A prompted Taeusch et al. to study the effects of other "sugars", including dextran, on the resistance of surfactant towards inhibitors [24]. For the currently available exogenous surfactant preparations that do not contain SP-A, dextran might be a useful addition in order to improve the maintainance of the structural and functional integrity of surface active subtypes. Curosurf also contains relatively little (≈1%) of SP-B and SP-C. As surfactant subtype conversion has also been shown to be related to loss of SP-B and surface activity in LA [5], this might explain why natural modified surfactants that are extracted from lung homogenate/lung lavage fluid with organic solvents are more prone to inhibition than "native" surfactant from amniotic fluid or lung lavage fluid [22]. In this respect, the development of new "designer surfactants" [21,50]with increased resistance to inactivation seems necessary for a more successful exogenous surfactant treatment of ARDS and related disorders like meconium aspiration syndrome. Such surfactants with recombinant proteins or peptide analogues of the surfactant apoproteins will be relatively expensive to produce. Dextran is cheap and it is easy to mix with surfactant. However, there is indication from animal work that the effects of dextran in vivo differ between various surfactants. In addition, dextran, due to its high osmolality, bears the risk of fluid accumulation in the lung, so that alternatives like polyethylene glycol [30], hyaluronic acid [30,51]or polymyxin B [52]are currently being investigated. The technique presented in our investigation might be useful for such studies as well.

## Conclusion

In conclusion, meconium causes alterations in the ultrastructural composition of the natural modified surfactant Curosurf that can be visualized and analyzed by TEM and stereology. These alterations resemble an increased SA/LA ratio, indicating that an increased conversion of surface active forms to inactive forms is involved in the pathomechanism of meconium-induced surfactant inactivation. These morphological alterations are paralleled by an increase in minimum surface tension. Dextran prevents these alterations and may therefore be a useful additive to exogenous surfactant preparations to preserve or reconstitute their structural and functional integrity, thereby improving their resistance to inactivation.

## Competing interests

The author(s) declare that they have no competing interests.

## Authors' contributions

MO conceived of and participated in the design of the study, carried out the transmission electron microscopic studies, supervised the stereological analysis, and drafted the manuscript. MS carried out the stereological analysis. GS mixed the components, carried out the surface tension measurements, and participated in the drafting of the manuscript. EH conceived of and participated in the design of the study, supervised the surface tension measurements, and participated in the drafting of the manuscript. All authors read and approved the final manuscript.
